# Pseudoepitheliomatous hyperplasia of the prepuce, a benign mimic of penile squamous cell carcinoma: a case report

**DOI:** 10.1186/s13256-026-05999-9

**Published:** 2026-04-07

**Authors:** Senthil Kumar Thiagarajan, Balaji Subramaniam, R. S. Rohitha, Dayakar Surati

**Affiliations:** Department of Urology, SRM Medical College Hospital & Research Centre, Chennai, Tamil Nadu India

**Keywords:** Pseudoepitheliomatous hyperplasia, Prepuce, Penile cancer, Differential diagnosis, Circumcision, Case report

## Abstract

**Background:**

Pseudoepitheliomatous hyperplasia (PEH) is a benign reactive proliferation of squamous epithelium that can closely mimic squamous cell carcinoma (SCC) both clinically and histologically. PEH commonly arises in response to chronic irritation, infection, or trauma. Its occurrence in the prepuce is extremely rare and poses significant diagnostic challenges.

**Case presentation:**

We report a case of a 75-year-old Indian male who presented with a 1-year history of progressive penile swelling, accompanied by obstructive voiding symptoms over recent weeks. Physical examination revealed a thickened, nodular, and edematous prepuce with phimosis, raising strong clinical suspicion of penile carcinoma. Incisional biopsy revealed features consistent with PEH, characterized by marked acanthosis, elongated and anastomosing rete ridges, and absence of cytological atypia. The patient subsequently underwent complete circumcision. Histopathological analysis of the excised specimen confirmed the diagnosis of PEH. The postoperative course was uneventful with complete resolution of symptoms.

**Conclusions:**

This case highlights the importance of including PEH in the differential diagnosis of suspicious penile lesions, particularly in elderly patients. Histopathological confirmation through biopsy is crucial to avoid unnecessary radical surgeries, such as partial or total penectomy. Early recognition and appropriate management can preserve function while ensuring optimal patient outcomes.

## Introduction

Pseudoepitheliomatous hyperplasia (PEH) is a benign, reactive proliferation of squamous epithelium that presents significant diagnostic challenges due to its remarkable resemblance to squamous cell carcinoma (SCC) both clinically and histopathologically. Histologically, PEH is characterized by irregular acanthosis, elongated and anastomosing rete ridges, and hyperkeratosis while crucially lacking the cytological atypia and basement membrane breach that characterize malignancy [1].

PEH typically develops as a reactive process secondary to chronic inflammatory dermatoses, trauma, chronic infections, or in association with neoplastic lesions. Common infectious etiologies include syphilis, tuberculosis, leishmaniasis, chronic candidiasis, and human papillomavirus (HPV) infection [2, 3]. It may also be associated with chronic inflammatory conditions, such as lichen sclerosus and chronic balanitis [4, 5].

While PEH has been reported in various anatomical sites, including the oral cavity, larynx, esophagus, and skin, its involvement of the male genitalia, particularly the prepuce, remains extremely rare. The prepuce represents a challenging anatomical site, where differentiation between benign and malignant lesions through clinical and pathological evaluation can be particularly difficult. This difficulty arises due to the prepice’s unique anatomical and physiological characteristics: it is a highly mobile, moist mucosal surgace prone to chronic irritation from smegma accumulation, poor hygiene, and recurrent microtrauma during erections or sexual activity. These factors can lead to persistent inflammation and epithelial proliferation that obscures the underlying pathology. In addition, complete phimosis often prevents adequate visualization and palpation of the glans and inner prepucial surface, limiting clinical assessment and increasing the risk of misdiagnosis. Elderly patients presenting with phimosis, nodular thickening, or verrucous preputial lesions are often presumed to have SCC, especially when accompanied by obstructive urinary symptoms or signs of chronic inflammation. This clinical presentation often leads to the consideration of aggressive surgical interventions, such as partial or total penectomy, if histopathological confirmation is not obtained prior to definitive management [6].

We present a rare case of a 75-year-old male with PEH of the prepuce, initially suspected to represent penile SCC, which was successfully managed following biopsy confirmation. This case underscores the critical importance of thorough histological assessment to ensure accurate diagnosis and appropriate treatment planning.

## Case presentation

A 75-year-old Indian male presented to our urology outpatient department with a 1-year history of gradually progressive penile swelling. Initially, the swelling was mild and painless; however, over the preceding 3–4 weeks, the patient developed increasing difficulty with urination, including straining, urinary hesitancy, and sensation of incomplete bladder emptying. He denied hematuria, urethral discharge, fever, weight loss, or other systemic symptoms. His past medical history was unremarkable, with no history of diabetes mellitus, hypertension, immunosuppression, or sexually transmitted infections.

**Fig. 1:**
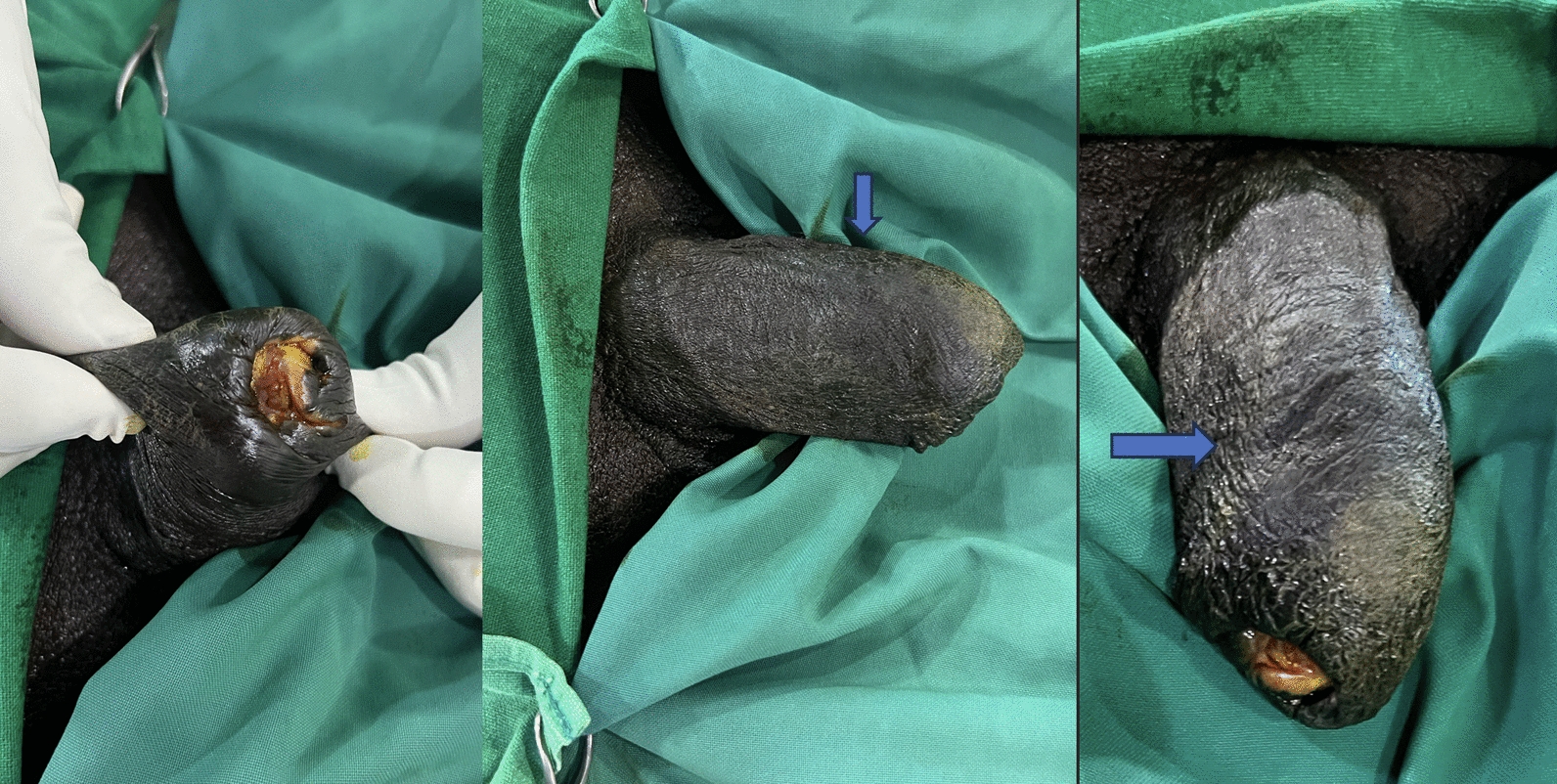
Clinical image showing nodular thickening of the prepuce with edema

Physical examination revealed a thickened, indurated, and nodular prepuce with significant edema. The glans penis was not visible due to complete phimosis. The surface of the lesion appeared verrucous and was non-ulcerative (Fig. 1). There was no active discharge or bleeding noted. Bilateral inguinal lymph node examination revealed no palpable lymphadenopathy. Based on these clinical findings, a provisional diagnosis of squamous cell carcinoma of the penis was made.

**Fig. 2:**
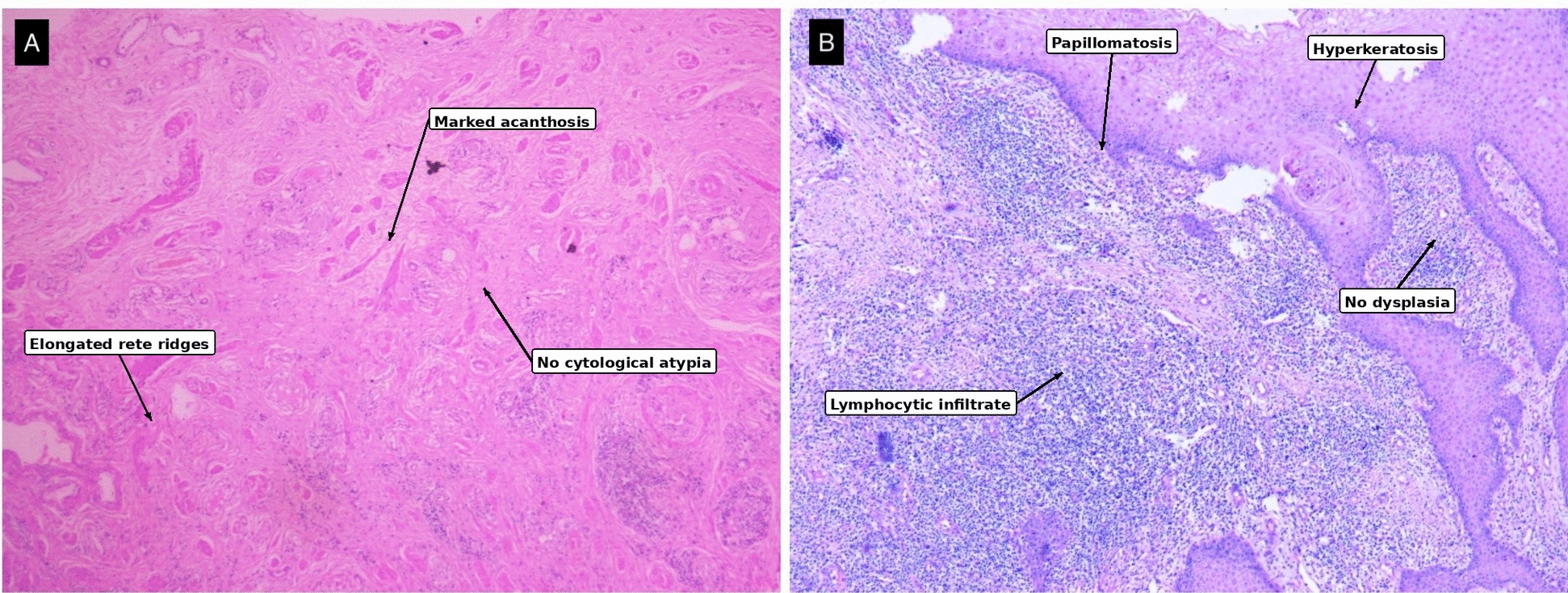
**A** Biopsy showing pseudoepitheliomatous hyperplasia with marked acanthosis and elongated rete ridges (H&E stain, 100 ×). **B** Post-circumcision specimen confirming stratified squamous epithelium lining with moderate acanthosis, papillomatosis, mild hyperkeratosis, focal ulceration with underlying fibrocollagenous stroma showing lymphocytic infiltrate and congested blood vessels. Suggestive of pseudoepitheliomatous hyperplasia with no evidence of dysplasia (H&E stain, 100 ×)

An incisional biopsy was obtained from the nodular preputial lesion under local anesthesia. Histopathological evaluation demonstrated marked pseudoepitheliomatous hyperplasia, characterized by irregular acanthosis, elongation, and branching of rete ridges, along with hyperkeratosis and parakeratosis. Importantly, there was no evidence of dysplasia, cytological atypia, or invasion into the underlying dermis (Fig. 2A).

Given the benign histopathological findings, the patient was counseled regarding the diagnosis and treatment options. He subsequently underwent complete circumcision under spinal anesthesia. Intraoperatively, the foreskin was found to be diffusely thickened and fibrotic but not adherent to the glans. The entire excised specimen was submitted for comprehensive histopathological evaluation, which confirmed the diagnosis of PEH without any malignant features (Fig. 2B).

The patient's postoperative recovery was uneventful. He was discharged on the second postoperative day and followed up at 1 and 3 months. At follow-up visits, he reported complete resolution of voiding symptoms with no signs of recurrence or complications.

## Discussion

PEH represents a benign condition that can present significant diagnostic challenges due to its striking clinical and histological resemblance to SCC. While the exact pathogenesis of PEH remains incompletely understood, it is widely accepted as a hyperplastic reaction to chronic irritation or inflammation [1, 2]. The histological appearance can be alarming and may closely mimic carcinoma, potentially leading to overtreatment if not carefully evaluated by experienced pathologists.

Histologically, PEH is characterized by pseudo-invasive growth of squamous epithelium with irregular, thickened, and branching rete ridges extending into the dermis. However, unlike SCC, PEH lacks cytologic atypia, atypical mitotic figures, or evidence of true basement membrane invasion [7]. Accurate diagnosis, therefore, relies on meticulous histological interpretation, often requiring consultation with experienced dermatopathologists to distinguish PEH from well-differentiated SCC.

PEH involving the prepuce is exceptionally rare, with only a few cases reported in the literature involving the penile region. To contextualize our findings, we reviewed similar cases of PEH of the prepuce or penile region, as summarized in Table 1.

**Table 1:** Literature review of PEH cases involving the prepuce

S.NO	Study	Year	Patient age	Clinical presentation	Associated conditions	Diagnostic method	Treatment	Outcome
1	Sarma and Weilbaecher [1]	1984	70	Thickened prepuce, voiding difficulty	None identified	Biopsy	Conservative excision	Full recovery, No complications
2	Chaux et al. [3]	2011	62	Nodular penile lesion, phimosis	Lichen sclerosus	Incisional biopsy	Circumcision	Complete resolution, No recurrence
3	Ficarra and Carlos [4]	2001	58	Verrucous preputial mass	Chronic balanitis	Biopsy	Local excision	No recurrence at 6 months
4	Yanofsky et al. [8]	2011	67	Ulcerated penile lesion	HPV infection	Biopsy	Circumcision	Symptom resolution, No malignancy
5	Current case	2025	75	Nodular, edematous prepuce, phimosis, obstructive voiding symptoms	None identified	Incisional biopsy, circumcision	Complete circumcision	Resolution of symptoms, No recurrence at 3 months

Studies were selected based on reported cases of PEH involving the prepuce or penile region, focusing on clinical presentation, diagnostic methods, and outcomes. Common associated conditions include chronic inflammatory states (e.g., lichen sclerosus, and balanitis) or infections (e.g., HPV). All cases emphasize the importance of histopathological confirmation to differentiate PEH from squamous cell carcinoma.

Previously reported cases have demonstrated associations between PEH and conditions, such as lichen sclerosus, chronic balanitis, or HPV infection, which may serve as chronic irritants initiating the hyperplastic response [4, 5, 8]. Interestingly, in our case, no identifiable predisposing factors such as infection, trauma, or dermatologic disease were evident, suggesting a possible idiopathic etiology, similar to the case reported by Sarma and Weilbaecher [1].

The clinical presentation can be highly misleading, as demonstrated in our patient, where the lesion presented as a thickened, nodular, verrucous prepuce with associated phimosis and voiding dysfunction. Such findings are highly suggestive of penile SCC, particularly in elderly patients. Without histological confirmation, such cases might undergo unnecessary and potentially devastating surgical procedures, such as partial or total penectomy, resulting in significant physical morbidity, functional impairment, and psychological distress [9].

Our case demonstrates that timely biopsy and comprehensive histopathological evaluation are absolutely critical in avoiding overtreatment. The lesion was successfully managed with complete circumcision, which provided both definitive tissue diagnosis and complete symptom relief. Postoperatively, the patient experienced complete resolution of his obstructive symptoms with no evidence of recurrence during follow-up.

This case contributes to the limited literature on PEH of the prepuce and emphasizes the critical importance of including PEH in the differential diagnosis of suspicious penile masses. It is imperative for clinicians, particularly urologists and dermatologists, to maintain awareness of this possibility in appropriate clinical contexts and to pursue histological confirmation before proceeding with radical surgical interventions.

From a clinical practice perspective, this case reinforces several important principles: first, the necessity of tissue diagnosis in suspicious penile lesions regardless of clinical appearance; second, the value of multidisciplinary consultation with experienced pathologists; and third, the importance of conservative surgical approaches when feasible, particularly after confirming benign pathology.

## Conclusion

Pseudoepitheliomatous hyperplasia of the prepuce represents a rare but benign condition that may closely mimic squamous cell carcinoma both clinically and histologically. Accurate diagnosis depends entirely on comprehensive histopathological evaluation, which can prevent overtreatment and unnecessary surgical morbidity. In elderly patients presenting with suspicious penile lesions, particularly those with nodular or verrucous features, PEH should be considered in the differential diagnosis. A systematic approach incorporating biopsy confirmation and, when appropriate, conservative excision can ensure excellent outcomes while preserving function and quality of life.

## Data Availability

Not applicable.

## Abbreviations

PEH: Pseudoepitheliomatous hyperplasia
SCC: Squamous cell carcinoma
HPV: Human papillomavirus
